# Juvenile prey induce antipredator behaviour in adult predators

**DOI:** 10.1007/s10493-012-9601-6

**Published:** 2012-08-25

**Authors:** Ângela Alves de Almeida, Arne Janssen

**Affiliations:** 1 Section Population Biology, IBED, University of Amsterdam, Science Park 904, 1098 XH Amsterdam, The Netherlands; 2 Department of Entomology, Federal University of Viçosa, Viçosa, Minas Gerais Brazil

**Keywords:** Phytoseiids, Thrips, Oviposition site selection, Role reversals, Predator–prey interactions, Counterattacking prey, *Amblyseius swirskii*

## Abstract

It is generally assumed that the choice of oviposition sites in arthropods is affected by the presence of food for the offspring on the one hand and by predation risk on the other hand. But where should females oviposit when the food itself poses a predation risk for their offspring? Here, we address this question by studying the oviposition behaviour of the predatory mite *Amblyseius swirskii* in reaction to the presence of its counterattacking prey, the western flower thrips *Frankliniella occidentalis*. We offered the mites a choice between two potential oviposition sites, one with and one without food. We used two types of food: thrips larvae, which are predators of eggs of predatory mite but are consumed by older predator stages, and pollen, a food source that poses no risk to the predators. With pollen as food, the predators preferred ovipositing on the site with food. This might facilitate the foraging for food by the immature offspring that will emerge from the eggs. With thrips as food, female predators preferred ovipositing on the site without thrips. Predators that oviposited more on the site with thrips larvae killed more thrips larvae than females that oviposited on the site without food, but this did not result in higher oviposition. This suggests that the females killed thrips to protect their offspring. Our results show that predators display complex anti-predator behaviour in response to the presence of counter-attacking prey.

## Introduction

Predators have important effects on the dynamics of their prey, either directly through the consumption of prey, or through indirect, non-lethal effects (Sih et al. 1985; Sih 1987; Kats and Dill 1998; Lima 1998). The direct effects affect only those prey stages that can be attacked and consumed, but do not affect invulnerable prey stages. However, predators can also indirectly affect invulnerable prey stages. For example when eggs, but not adults, are vulnerable to predation, adult female prey can avoid oviposition at sites that are accessible to predators, thus reducing the risk of predation on their offspring (Resetarits and Wilbur 1989; Mappes and Kaitala 1995; Pallini et al. 1999; Binckley and Resetarits 2002; Faraji et al. 2002; Reguera and Gomendio 2002; Binckley and Resetarits 2003; Murphy 2003; Nomikou et al. 2003; Eitam and Blaustein 2004; Montserrat et al. 2007; Carrasco and Kaitala 2009; Hirayama and Kasuya 2009; Choh and Takabayashi 2010; Choh et al. 2010; van der Hammen et al. 2010a, b).

Predator–prey interactions become even more complicated when there are role reversals between predators and prey. Such role reversals, where vulnerable, young, small predators can be killed by large prey, are common in nature (Saito 1986; Barkai and McQuaid 1988; Polis et al. 1989; Palomares and Caro 1999; Janssen et al. 2002; Magalhães et al. 2005b). In such cases, antipredator behaviour is not only found in prey in response to the presence of predators or predator cues, but also in predators in response to prey that are capable of counterattacking (Faraji et al. 2001, 2002; Janssen et al. 2002; Magalhães et al. 2005b). Here we study one type of antipredator behaviour in a predatory mite in response to the presence of prey that attack the eggs of the predatory mite.

The prey used was the omnivorous western flower thrips *Frankliniella* *occidentalis* (Thysanoptera: Thripidae), one of the most important pests worldwide of crops such as cucumber, tomato, sweet pepper, rose and begonia. The predatory mite *Amblyseius swirskii* (Athias-Henriot) is a new control agent for Western flower thrips, tobacco whitefly (*Bemisia tabaci*), greenhouse whitefly (*Trialeurodes vaporariorum*), broad mites (*Polyphagotarsonemus latus*), chilli thrips (*Scirtothrips dorsalis*) and possibly also tomato russet mite (*Aculops lycopersici*) (Nomikou et al. 2001, 2002, 2004; Messelink et al. 2006; Messelink et al. 2008; Wimmer et al. 2008; Arthurs et al. 2009; Park et al. 2010; van Maanen et al. 2010; Calvo et al. 2011). The predatory mites attack young, small thrips larvae, which usually feed on plant tissue. The thrips larvae, however, can defend themselves against such attacks by hitting the predators with violent blows with their abdomen and by producing droplets of fluid, which, upon contacting the predator, cause it to stop the attack (Bakker and Sabelis 1989; de Bruijn et al. 2006). Thrips adults and larvae can also counterattack predatory mites; they kill and consume eggs of several species of predatory mites (Janssen et al. 2003), including *A. swirskii* (A. Janssen personal observation). Thrips larvae of the strain used here benefit from consuming predator eggs because it supplements their diet (Janssen et al. 2003; Magalhães et al. 2005a), but the killing of the eggs also deters adult predatory mites, thus reducing the predation risk of the thrips larvae that killed the eggs (Janssen et al. 2002; Magalhães et al. 2005b).

In this paper, we evaluated the response of *A. swirskii* to the presence of dangerous, counterattacking thrips larvae. We expected the predators to oviposit on patches with non-prey food (pollen) to facilitate the foraging of their juvenile offspring, but to oviposit away from patches with dangerous prey.

## Materials and methods

*Amblyseius swirskii* was reared in the laboratory with pollen of *Typha latifolia* L. as food on plastic arenas (8 × 15 cm) placed on a wet sponge in a plastic tray containing water (Nomikou et al. 2003). Thrips (*F. occidentalis*) were reared in climate boxes on cucumber plants cv. Aviance RZ, which were grown from seeds in soil in plastic pots and kept in a walk-in climate room, free of herbivores. All experiments were performed in a climate room at 25 °C, 70 % RH and 16/8 photoperiod.

The experimental arenas consisted of two leaf discs connected with a small strip of leaf vein, the entire arena was cut from a cucumber leaf (Janssen et al. 2002). The two discs (diam. 36 mm), connected by their shared midrib (6–7 cm long, 3 mm wide), were floating on water-soaked cotton wool inside a Petri dish. In experiments with dangerous food, five young 1st-instar thrips larvae were placed on one leaf disc, whereas the disc on the other side was left clean. In experiments with safe food, a tiny amount of *Typha* pollen was added to one leaf disc and the other disc was left clean. Both food types are readily consumed by adult female *A. swirskii*, resulting in comparable oviposition rates (Nomikou et al. 2001; Messelink et al. 2008). Gravid female predatory mites were placed on the leaf vein in between the two discs. One day after introducing the female, we recorded the number of eggs on each disc, the number of thrips eaten and the side on which the female was found. We tested a total of 32 adult female predators with dangerous food and 21 females with safe food.

Although it would, in theory, have been interesting to offer the predatory mites a choice between a disc with pollen and a disc with thrips larvae, we refrained from doing this for practical reasons. Because thrips larvae also feed on pollen and, when doing so, feed significantly less on the eggs of predatory mites (Janssen et al. 2003), such a treatment would also reduce the predation risk of the eggs.

### Statistical analyses

The distribution of eggs was compared between treatments (pollen or thrips larvae) with a generalized linear model (GLM) with a binomial error distribution (Crawley 2007). The total amount of eggs produced was compared between treatments with a GLM with a Poisson error distribution. Within treatments, oviposition on the disc with food (either pollen or thrips larvae) was compared with oviposition on the clean disc with the Wilcoxon matched-pairs signed-rank test (Siegel and Castellan 1988). We tested for an effect of the number of thrips larvae eaten on total oviposition and on the distribution of eggs over both discs with GLMs with a Poisson error distribution and a quasi-binomial error distribution, respectively (Crawley 2007). All statistical analyses were done using R (R Development Core Team 2010).

## Results

**Fig. 1:**
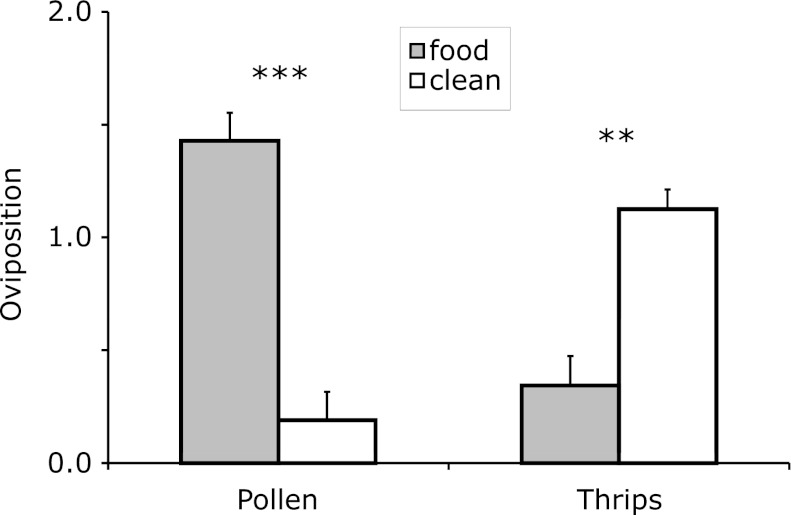
The choice of oviposition site by the predatory mite *Amblyseius swirskii*. Female predators were offered a choice between a leaf disc with food (pollen or thrips larvae) and a clean leaf disc. The two discs were connected with a bridge consisting of the leaf vein. With pollen as food, female predators preferred ovipositing on the disc with pollen, with thrips, they preferred ovipositing on the clean disc. *Asterisks* *above* the *bars* indicate significant preference, Wilcoxon matched-pairs signed-rank test **P < 0.005; ***P < 0.001

Depending on the type of food, adult female *A. swirskii* preferred ovipositing near the food or on the disc without food (Fig. 1; GLM: deviance = 36.5, df = 1,51, *P* < 0.0001). The females preferred ovipositing on the disc with pollen compared to the clean disc (Wilcoxon matched-pairs signed-ranks test: V = 166.5, *P* = 0.0003; Fig. 1). This might facilitate the foraging for food by the immature offspring that will emerge from the eggs. In contrast, female predators oviposited more on the clean disc than on the disc with dangerous thrips larvae (V = 373, *P* = 0.003; Fig. 1), suggesting that they avoided ovipositing near prey that can attack and kill the predator’s eggs.

The predators produced on average 1.62 (±0.11, SEM) eggs in the experiment with pollen and 1.47 (±0.10) in the experiment with thrips larvae. This difference was not significant (GLM: deviance = 0.186, df = 1,51, *P* = 0.67), confirming that the food types did not differ much in quality (Nomikou et al. 2001; Messelink et al. 2008).

**Fig. 2:**
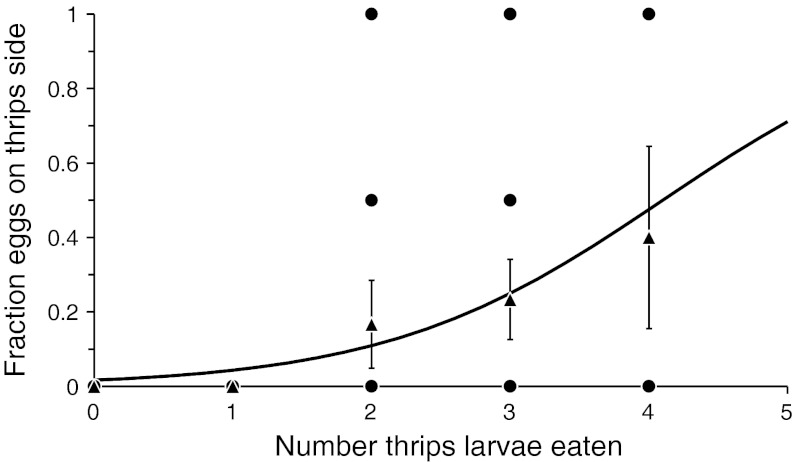
The fraction of eggs oviposited by the predatory mite *Amblyseius swirskii* on a leaf disc with thrips larvae, a dangerous prey, as a function of the number of thrips larvae that were eaten by the predator. *Circles* give the raw data; *triangles* show average fractions plus standard errors and are included for ease of interpretation. The curve was fitted with a GLM with a binomial error distribution: y = 1/(1 + e^41−x^)

There was no significant effect of the number of thrips larvae consumed on the number of eggs produced (GLM with Poisson errors: deviance = 0.0006, df = 1,30, *P* = 0.98), but there was a significant effect of the number of thrips larvae killed on the fraction of eggs oviposited on the disc with thrips larvae (Fig. 2; GLM with quasi-binomial errors: F_1,30_ = 4.82, *P* = 0.036).

## Discussion

It is known that *F. occidentalis* larvae are capable of discriminating between the eggs of harmless and dangerous predators, and kill significantly more eggs of the dangerous predator (Janssen et al. 2003). This increased killing deters adult predators, resulting in a reduction of the predation risk of the vulnerable thrips larvae (Janssen et al. 2002). Here, we show that *A. swirskii* anticipates such possible counterattacks by dangerous prey by ovipositing away from sites where dangerous prey occur. Because predatory mites have to feed to produce an egg, they have to commute between the disc where they oviposit and the disc where they feed on thrips larvae. Similar behaviour was found for the predatory mite *Iphiseius degenerans*, which commutes between flowers of sweet pepper plants, where it feeds on thrips larvae and pollen, and leaves, where it oviposits in domatia, where the eggs are safe from predation by thrips (Faraji et al. 2001, 2002). This behaviour has the disadvantage that the offspring hatching from the eggs do not immediately have access to food, but have to search for it. This may increase the starvation risk of the offspring, which may be an important cost of this antipredator behaviour. Moreover, the females have to spend time and energy in commuting between sites with food and oviposition sites (Faraji et al. 2001). Apparently, these disadvantages are outweighed by the reduced predation on predator eggs.

We do not know which cues the predators used to assess the potential risk of predation for their offspring. Often, antipredator behaviour is mediated by chemical cues, which are released or left behind by natural enemies and prey (Dicke and Grostal 2001; de Bruijn et al. 2006). Here, the prey represent the danger, but at the same time, they are an important food source for the predator. This suggests that the response of the predator to the cues associated with prey depends on the physiological state of the predator: they are attracted by the cues when hungry, but when satiated and ready to oviposit, they avoid these cues. All females used here contained an (almost) full-grown egg at the start of the experiment, and produced one to two eggs (one female produced 3 eggs), hence they will all have changed physiological state at least once during the experiment.

Oviposition in predatory mites is closely linked to food intake (Sabelis 1986). However, there was no significant effect of the number of thrips larvae consumed on the number of eggs produced. This suggests that thrips density did not limit egg production. Furthermore, there was a significant effect of the number of thrips killed on the fraction of eggs oviposited on the disc with thrips larvae (Fig. 2). These two results together show that the predators that oviposited more on the disc with thrips larvae killed more thrips larvae, but this did not result in higher oviposition. Perhaps the predators killed thrips in order to defend their eggs against attacks by thrips, as was found for another species of predatory mite (Magalhães et al. 2005b). It is also possible that the adult females killed thrips larvae to allow their immature offspring to scavenge on them. The results suggest that there are two strategies to reduce killing of predator offspring by counterattacking prey: (1) avoid counter-attack by killing more prey, thus making the site safe for the offspring; (2) choose a safe place without counterattacking prey to lay eggs. Under the first strategy, the parental care displayed by predator in response to counterattacking prey results in more prey being killed, and this raises the question of why prey would counterattack in the first place. Under the second strategy, killing eggs of predators is advantageous for the thrips larvae because predators that move to another site to oviposit do not necessarily return, especially not when food is also obtainable elsewhere, and this reduces the predation risk of the thrips larvae (Janssen et al. 2002). Future studies should verify that the two strategies outlined above indeed occur in populations of *A. swirskii* and whether these are fixed strategies, i.e. whether some individuals always avoid ovipositing near thrips larvae whereas others always defend their eggs by killing more thrips larvae. Alternatively, these strategies could be conditional, depending on the initial interactions between the predatory mites and their dangerous prey.

As noted by Lima (1998), virtually all animals are both predators (because they consume other organisms, including plants) as well as potential prey. This may pose opposing selection pressures on their behaviour. Our experimental system is an excellent example of this: ovipositing close to a food source may help offspring in detecting food, but if the food is dangerous, it poses a risk at the same time. We show that the predator adjusts its oviposition behaviour according to the predation risk of its offspring. This antipredator may affect density-mediated effects substantially, but such effects are often difficult to quantify (Abrams 2008). Our results show that predator-prey interactions are much more complex than often assumed when sizes of prey and predators overlap and when this results in reversals of their ecological roles.
